# Colorectal anastomotic leakage: a narrative review of definitions, grading systems, and consequences of leaks

**DOI:** 10.3389/fsurg.2024.1371567

**Published:** 2024-05-02

**Authors:** Olivia Rennie, Manaswi Sharma, Nour Helwa

**Affiliations:** ^1^ Department of Clinical Affairs, FluidAI Medical (Formerly NERv Technology Inc.), Kitchener, ON, Canada; ^2^ Faculty of Medicine, University of Toronto, Toronto, ON, Canada

**Keywords:** anastomosis, anastomotic leak, colorectal surgery, definitions, grading systems, complications, consequences

## Abstract

**Background:**

Anastomotic leaks (ALs) are a significant and feared postoperative complication, with incidence of up to 30% despite advances in surgical techniques. With implications such as additional interventions, prolonged hospital stays, and hospital readmission, ALs have important impacts at the level of individual patients and healthcare providers, as well as healthcare systems as a whole. Challenges in developing unified definitions and grading systems for leaks have proved problematic, despite acknowledgement that colorectal AL is a critical issue in intestinal surgery with serious consequences. The aim of this study was to construct a narrative review of literature surrounding definitions and grading systems for ALs, and consequences of this postoperative complication.

**Methods:**

A literature review was conducted by examining databases including PubMed, Web of Science, OVID Embase, Google Scholar, and Cochrane library databases. Searches were performed with the following keywords: anastomosis, anastomotic leak, colorectal, surgery, grading system, complications, risk factors, and consequences. Publications that were retrieved underwent further assessment to ensure other relevant publications were identified and included.

**Results:**

A universally accepted definition and grading system for ALs continues to be lacking, leading to variability in reported incidence in the literature. Additional factors add to variability in estimates, including differences in the anastomotic site and institutional/individual differences in operative technique. Various groups have worked to publish guidelines for defining and grading AL, with the International Study Group of Rectal Cancer (ISGRC/ISREC) definition the current most recommended universal definition for colorectal AL. The burden of AL on patients, healthcare providers, and hospitals is well documented in evidence from leak consequences, such as increased morbidity and mortality, higher reoperation rates, and increased readmission rates, among others.

**Conclusions:**

Colorectal AL remains a significant challenge in intestinal surgery, despite medical advancements. Understanding the progress made in defining and grading leaks, as well as the range of negative outcomes that arise from AL, is crucial in improving patient care, reduce surgical mortality, and drive further advancements in earlier detection and treatment of AL.

## Introduction

Colorectal surgery is defined as a surgical procedure involving large and/or small bowel resection and reconstruction. Classic colorectal procedures are described by the level of resection (proximal, middle, or distal) and the method of reconstruction utilized (i.e., creation of stoma vs. anastomosis). Colorectal procedures are conducted to help treat various pathologies including (but not limited to) colorectal cancer (CRC), mechanical bowel obstruction, recurrent diverticulitis, familial adenomatous polyposis, and inflammatory bowel diseases (IBD) such as ulcerative colitis, Crohn's disease, and indeterminate colitis (1). Additionally, colorectal procedures may be done in response to injury, ischemic colitis, refractory constipation, rectal prolapse and proctological disorders (1). Colorectal surgery is not without its risks, many of which can be severe and life-threatening. As an example, surgical site infections (SSIs) are a common in-hospital infection (and the most common post-operative complication associated with colorectal procedures), with prevalences estimated at 2.4%–21.6% of patients undergoing colorectal surgery (2). In addition to patient suffering, this complication carries high negative economic impacts, increases morbidity, and elevates the likelihood of readmission, extended hospital stay, and potentially, death (2). Postoperative sepsis following colorectal surgery is another serious risk, estimated to occur in >1% of elective procedures, and >4% of non-elective procedures, respectively (3). With mortality rates estimated to be approximately 25%, sepsis remains a significant concern for patients and surgeons alike when performing procedures such as gastrointestinal surgery (3). While the importance of complications like SSIs and sepsis cannot be understated, this publication focuses specifically on another deadly and pervasive postoperative complication: anastomotic leakage (AL). In some cases, AL may occur concurrently with other complications, such as SSIs and sepsis (among others), as will be described below.

Most colorectal procedures involve the resection of a target pathology followed by the restoration of gastrointestinal continuity through the creation of an anastomosis. An anastomosis can be defined as a hand-sewn or stapled connection between two tubular structures (4). As with any surgical intervention, the creation of an anastomosis does not come without risk. Some of the main postoperative complications associated with the creation of an intestinal anastomosis following colorectal surgery include surgical site infection, bleeding, stenosis, fistula formation, ileus, and AL/dehiscence. If not managed properly, such complications can lead to sepsis, septic shock, or even death. Of those complications, AL is considered a major source of morbidity and mortality with rates equivalent to 20%–35% and 2%–16.4% respectively (5).

Despite advances in surgical techniques, the incidence of AL has not changed significantly in recent decades and was reported in literature to vary from 2.8% to as high as 30% (5). A variety of different patient factors have been associated with increased leak risk, including increased age, male sex, smoking, alcohol use, high American Society of Anesthesiologists (ASA) scores, operating time, nutritional status, mechanical bowel preparation, and steroid use (6–8). AL is associated with additional intervention, prolonged hospital stays, and hospital readmission. The objective of this narrative review is to summarize the changing landscape of defining and grading ALs, as well as the significant consequences of leaks at both the patient and healthcare systems level.

## Methods

The most recent evidence from various databases was used to inform this narrative review, including PubMed, Web of Science, OVID Embase, Google Scholar, and Cochrane library databases. Literature searches were performed using the following keywords: anastomosis, anastomotic leak, colorectal, surgery, grading system, complications, risk factors, and consequences. All articles and relevant reviews that were retrieved underwent manual assessment for other potentially relevant publications. No restrictions were placed on article type. Inclusion was determined by the authors, aiming to include a broad, unbiased range of relevant and recent studies.

## Definitions and AL grading systems

Anastomotic leakage is used synonymously with anastomotic leak, anastomotic insufficiency, anastomotic failure, anastomotic defect, anastomotic breakdown, suture insufficiency, suture line disruption, and anastomotic dehiscence. In attempting to accurately capture the incidence of ALs—and by extension, develop better detection and treatment—defining both the complication itself, and various degrees of severity, is imperative. Unfortunately, a universally accepted definition and grading system continue to be lacking, leading to variability in the reported incidence of AL (which ranges in the literature widely—from 2.8%–30%) (5, 9, 10). Thus, the reported incidence continues to vary depending on the clinician/research group's definition of leakage. Additional factors adding to the variability in estimates include differences in the anastomotic site, institutional and individual differences in operative technique, preoperative factors, intraoperative factors, and postoperative factors (11, 12).

Various groups such as the United Kingdom Surgical Infection Study Group (SISG) (13) and the International Study Group of Rectal Cancer (ISGRC/ISREC) (12) published guidelines for defining and grading AL. The Clavien-Dindo (CD) surgical complication severity scale was also proposed for the grading/classification of AL (Grade I, II, IIIa, IIIb, Iva, Ivb, V). While each of these guidelines is an important starting place, none have yet been widely accepted. In an early review conducted by Bruce et al. (14), 29 different definitions of lower gastrointestinal leakage were reported across 49 studies (14). Further, consensus-based surveys conducted by Adams et al. and Van Rooijen et al. in 2013 and 2017, respectively, continued to demonstrate no uniform definition of AL (and beyond this, that significant heterogeneity still exists) (15). A more recent systematic review of 2,938 abstracts and 1,382 full-text articles showed that only 347 articles highlighted a definition of AL—and that this definition varied significantly across studies (16). The lack of a widely accepted definition results in highly variable incidence rates and prevents the proper comparison of data across various studies and centers.

### Clinical grading systems

Due to the urgent need of for a broadly accepted definition, the Italian Society of Surgery (SIC) (2020) and other reputable study groups published multiple international studies utilizing the Delphi method to establish a recommended general definition of AL (16–18). Today, the ISGRC/ISREC definition is the most recommended universal definition for colorectal AL (see Tables 1, 2).

**Table 1:** Definition and leak grading system: United Kingdom Surgical Infection Study Group – 1991 (13).

Verbatim definition	“A leak of luminal contents from a surgical join between two hollow viscera.”
Clinical leak	Defined as the leak of luminal contents through the wound or at the drain site, or the collection of such content at the anastomosis resulting in one of the following symptoms/findings: fever, abscess, septicaemia, metabolic disturbance and/or multiple-organ failure
Subclinical leak	Usually detected via imaging; defined as the leak of luminal contents from an anastomosis into an adjacent localised area. Patients that present with subclinical leaks have no clinical symptoms or signs of leakage

**Table 2:** Definition and leak grading system: International Study Group of Rectal Cancer – 2010 (12).

Verbatim definition	“A communication between the intra- and extraluminal compartments owing to a defect of the integrity of the intestinal wall at the anastomosis between the colon and rectum or the colon and anus. Because of its similar clinical impact, a leakage originating from the suture or staple line of a neorectal reservoir (e.g., J-pouch or transverse coloplasty) should also be considered as an anastomotic leakage. Furthermore, we recommend considering a pelvic abscess in the proximity of the anastomosis as anastomotic leakage”.
Grade A	Defined as an AL requiring no active therapeutic intervention. This grade of anastomotic leakage is not associated with clinical signs/symptoms or abnormal laboratory tests. Radiological evaluation may show a small, contained leak. Contents from the drain (if present) are usually serous however, some patients may present with turbid or feculent contents.
Grade B	Defined as an AL requiring active therapeutic intervention (administration of antibiotics, interventional drainage, or transanal drainage). Such leaks are usually managed without operative reintervention. Clinical symptoms associated with Grade B leaks include mild/moderate discomfort with abdominal/pelvic pain, leukocytosis, elevated serum C-reactive protein (CRP), and turbid/purulent rectal or vaginal discharge. Radiological evaluation conducted on these patients show leakage of the endoluminally administered contrast agent at the anastomosis. CT scans may also reveal abscess formation.
Grade C	Defined as an AL requiring relaparotomy. This can include performing a Hartmann's procedure or creating a protective ileostomy. Clinical symptoms associated with Grade C leaks include severe discomfort, leukocytosis, elevated serum CRP, abdominal pain, fever, purulent/fecal drainage, and signs of sepsis/peritonitis (abdominal wall rigidity, tenderness to palpation, tachycardia, hemodynamic instability, leukopenia, hypothermia, organ failure, etc.). Radiological evaluation conducted on these patients reveals considerable leakage at the anastomotic site with fluid collection(s).

## Consequences of leaks

Colorectal AL is considered the bane of intestinal surgery and is one of the most feared complications due to the associated clinical and economic burden. AL can impose a significant burden on patients, healthcare providers, and hospitals.

### Increased morbidity

A large number of research articles report that AL results in high morbidity. A prospective multicenter international study conducted by the European Society of Coloproctology in 2015 reported a 30-day morbidity rate of 38.0% in AL patients who underwent a right hemicolectomy or ileocecal resection (19). Another study conducted by Alves et al. reported an overall morbidity rate of 35% in AL patients (20). Additionally, McArdle et al. and Branagan et al. reported that the 30-day mortality is 12.1% and 24.7% higher in AL when contrasted to non-anastomostic leak (nAL) patients, respectively (21, 22).

### Increased mortality

Overall, the reported mortality rate in patients that present with AL varies between 6% and 39% (23). A study conducted by Bakker et al. showed that the mortality rate in patients with AL was 13.3% higher than the reported rate in their non-leaking counterparts (24). Another study compromised of 28,271 patients reported that the mortality rate was 16% higher in AL patients (25). A meta-analysis with a cohort of 154,981 patients revealed that AL has a significantly negative impact on overall survival (26).

### Increased rate of secondary postoperative complications

AL (specifically Grade B and C leaks) is associated with the need for a change in patient management and in particular, the need for antibiotic administration, interventional drainage, endoscopic management, intensive care unit (ICU) admission, increased length of stay or reoperation to prevent sepsis and peritonitis (27). Such interventions can have a devastating impact on the patient's postoperative course. Perioperative analysis by Kube et al. showed that AL is associated with secondary postoperative complications (28). These include pneumonia, pulmonary complications, cardiac complications, renal complications, wound infection, abscess formation, enterocutaneous fistula, complete rupture of the operation wound, sepsis, peritonitis, and multi-organ failure. Kube et al. showed that the rates of the above complications were significantly higher (*p* < 0.05) in AL patients when contrasted to nAL with an incidence of 62.7% in AL and 19.9% in patients without AL (28). Additionally, a retrospective analysis of data from more than 600 US hospitals revealed that AL patients had a higher postoperative infection rate (0.8–1.9 times increase) compared with patients without leaks (29). Recently, new biomarkers have been under investigation, with the aim to provide earlier detection of sepsis associated with complications like AL, as well as SSIs. For example, two separate studies, both published in 2023, provided evidence that the enzyme butyrylcholinesterase (BChE) could serve value as a prognostic marker of infectious complications after colorectal procedures. In a publication by Mulita et al. based on 402 patients, serum BChE was found to be significantly lower in patients with sepsis on postoperative days 3 and 5 compared to patients without septic complications (*p* = 0.015 and *p* = 0.029), respectively (30). Verras et al. investigated serum BChE as a predictive biomarker for SSI in colorectal patients, finding that BChE levels on the 1st and 3rd postoperative day were significant independent predictors of SSI (*p* < 0.001) (31). While still in the early days of identifying key biomarkers that could lead to earlier intervention of postoperative complications associated with AL and SSI, there is promise that continued study of these markers might even provide earlier leak grading, helping to further guide clinical intervention.

### Increased risk of permanent stoma

The risk of permanent stoma after clinical leakage is reported in literature to vary between 10% and 100% (32). A retrospective analysis of 1,442 patients revealed that the overall rate of permanent stoma among patients with AL was measured at 65% (33). Such intervention can highly impact a patient's overall satisfaction and quality of life (34).

### Higher reoperation rate

According to literature, AL leads to secondary complications and results in an increased risk of reoperation by more than 10-fold (35). One study of 600 patients reported a significantly higher reoperation rate of 91.7% vs. 5.4% in patients that presented with a leak and those that did not respectively (10). Other research articles reported a reoperation rate of 50%–60% in patients with AL (36).

### Poor health-related quality of life (HRQoL) and functional outcomes

AL is a severe complication associated with ICU admission, increased length-of-stay (LOS), and reoperation. Such interventions can have a profound impact on a patient's quality of life. A case-matched study conducted by Marinatou et al. utilized several validated questionnaires to compare the short-term and long-term HRQoL in AL and non-AL patients, assessing HRQoL at baseline (time of surgery), 3, 6, and 12 months postoperatively (37). 12 months after surgery, the Medical Outcomes Study (MOS) Short Form, European Organization of Research and Treatment of Cancer Quality of Life Questionnaire (EORTC QLQ-C30), and Gastrointestinal Quality of Life Index Questionnaire (GIQLI) revealed that AL patients had a significant reduction in physical function, social function, emotional function, and general health perception (37). AL patients also reported role limitations due to physical health and emotional problems. Another study by Cristofaro et al. revealed that leakage was an independent predictor of quality of life and highly impacted the patient-surgeon satisfaction level (38). Here, 116 patients answered three versions of the EORTC, assessing generic (EORTC QLC-C30), disease-specific (EORTC QLC-CR29), and quality of life and treatment satisfaction (EORTC IN-PATSSAT32) at admission, and 1 and 6 months after surgery. Significantly worse outcomes, including postoperative psychiatric complications, were found in AL patients, impairing quality of life (*p* = 0.004) (38). Patients with AL of lower rectal anastomoses have also shown a 33% reduction in their neorectal capacity, significant tenesmus, and incontinence. Such impaired anorectal function had severe implications on the patient's HRQoL as identified by Nesbakken et al. (39).

### Extended length-of-stay (LOS)

Length of stay has been used as an indicator of the quality of care. Enhanced Recovery After Surgery (ERAS) protocols have been developed to enhance postoperative recovery and reduce LOS without affecting patient outcome. Multiple research articles reported an increase in LOS due to AL. Retrospective analysis conducted on data collected from more than 600 hospitals throughout the US showed that the mean LOS of patients with AL was 2.4 times higher (23 vs. 9.7) than patients without leakage (29). Another retrospective study including 8,597 patients that underwent elective resection showed that the mean LOS for AL patients was 2.7–2.9 times higher than non-leaking patients (40). A more recent analysis of 337 patients who underwent low anterior resection (LAR) in a Brazilian center revealed that the average length of hospitalization for AL patients was 39.6 days and 7.5 days for non-leaking patients (41). This is equivalent to a 5.3-fold increase in the average length of patient stay. Lastly, a study conducted by Hammond et al. reported that the total LOS of AL per 1,000 patients was 9,500 days longer in AL patients (LOS was measured at 26,300 days in AL patients vs. 16,800 days in nAL) (29).

### Increased hospitalization cost

AL is associated with a higher total cost due to prolonged hospitalization, the need for further diagnostic workup, and re-intervention. In fact, a study conducted by Braga et al. revealed that an AL is regarded as one of the most expensive postoperative complications as shown in a single-center randomized trial (42). The overall cost of AL per patient was reported to vary between €37,609–71,940. 60% of such added cost was attributed to the increased LOS, while 40% of the cost was tied to the resources used to diagnose and treat the AL (43). Other studies conducted by Ashraf et al. and Hammond et al. to evaluate the burden of AL reported that the annual direct healthcare cost associated with AL in the UK alone was equivalent to £1.1–3.5 million, while the mean cost of AL in the US was calculated at over $72,905 per patient (29, 44, 45). This was 2.9-times higher than the cost observed in patients without AL. Finally, according to a study published by Hammond et al. the difference in cost of AL and nAL per 1,000 patients was equivalent to $28.6 million (29).

### Increased readmission rates

Reduced hospital readmissions are used as a marker of the quality of care provided by hospitals. Readmissions are associated with an economic burden, poor patient satisfaction, poor patient outcomes, and have also recently been tied to hospital reimbursement (46). AL is regarded as one of the most common causes for postoperative readmission, and therefore, early identification and treatment is paramount to reducing readmission in these patients. A retrospective analysis of 6,174 patients revealed that the 30-day readmission rate of patient with AL was equivalent to 29% whereas the readmission rate in patients that did not present with leakage was measured at 13% (29). Furthermore, the overall readmission cost and length of stay upon readmission was 1.9 times and 1.8 times higher respectively for AL patients vs. patients without a leak (47).

### Increased ICU admission

AL is associated with life threatening intra-abdominal peritonitis, sepsis, and multiorgan failure requiring the need for ICU admission for organ support in the postoperative period (48). In fact, one study reported that the unplanned ICU admission rate associated with AL was 30.3% (49). Another study of 323 patients highlighted that admission to intensive care was required in 22.9% of patients that presented with leakage (50).

### Poor oncological outcome and poor prognosis

In patients who underwent resection for colorectal cancer, an association between AL and increased risk of cancer recurrence and poor oncologic prognosis has been noted in the literature. It is hypothesized that the elevated inflammatory markers [such as C-reactive protein (CRP)] associated with AL stimulate tumour proliferation and neoangiogenesis which leads to higher recurrence rates and reduces the overall survival/disease-free survival (51). One study reported a 9.2 increase in local recurrence rate in patients that presented with leakage (52). Similarly, Merkel et al. analyzed the data from 940 colorectal patients and concluded that the rate of locoregional recurrence in AL patients was 9.5% higher than in non-leaking patients (53). Another study by Law et al. highlighted that AL is regarded as an independent factor for an increased local tumor recurrence rate after curative resection in colorectal patients (hazard ratio: 2.55, 95% CI: 1.07–6.06, *p* = 0.034) (54). A much larger meta-analysis including 78,434 colorectal cancer patients revealed similar results showing that AL was associated with increased local recurrence (RR = 1.90) after curative resection (55). Evidence regarding the impact of AL on distal recurrence is mixed, with some studies finding adverse effects of AL on recurrence risk (e.g., Denost et al., Krarup et al.), while others showing no significant impact (e.g., Bashir Mohamed et al., Koedam et al.). Denost et al. conducted a study based on oncological data from 449 patients, finding distant recurrence-free survival was negatively influenced by AL (*p* = 0.046) (56). Krarup's et al. separate study based on 744 patients registered in the database of the Danish Colorectal Cancer Group, Danish Pathology Registry, and National Patient Registry found that distant recurrence developed more frequently after AL (adjusted HR = 1.42; 95% CI: 1.13–1.78; *p* = 0.003) (57). Conversely, Bashir Mohamed's et al. (58) systematic review and meta-analysis based on 69,047 total patients (2,555 with AL) demonstrated no significant effects on either local recurrence (RR = 1.16; 95% CI: 0.84–1.59) or distant recurrence (RR = 1.44; 95% CI: 0.52–3.96) (58). Later, Koedam et al. also found that AL was not a significant variable for distant recurrence (HR = 1.02; 95% CI: 0.33–3.21) (59).

### Limitations of the present study

While every effort was made to include all possible literature within this narrative review, it should be noted that this study is not a systematic review. During assessment of the literature, we aimed to be as thorough and detailed as possible in the search process, thus providing a clear consensus on the topics included within this publication. We focused on mitigating the natural limitations of narrative reviews by including a variety of different sources, providing as complete and unbiased a picture as possible. It was ensured that sources from a variety of different settings and patient populations were included in the study results. As an additional note, the fact that AL is an underreported postoperative complication also creates challenges for full assessment of its impacts. Underreporting may lead to challenges in drawing complete conclusions—especially concerning leaks which may be managed conservatively, have minimal clinical consequences, or are difficult to detect/diagnose (e.g., Grade A leaks).

## Conclusion

Despite continued advancements in clinical medicine, defining, detecting, and treating ALs continues to pose significant challenges. Creating a universally accepted grading system remains elusive, though important strides forward have been made to develop approaches to conceptualize and define leaks of varying severity. Understanding grading systems is essential in both clinical and research settings, where issues in properly identifying and classifying leaks creates major limitations for diagnosis, estimates of incidence, and appropriate patient classification in research analyses.

Beyond challenges at the diagnostic level, the severe consequences of leaks cannot be understated. With high morbidity and mortality, increased rate of secondary postoperative complications, higher reoperation rates, and extended length of stay (among others), burdens remain for not only the patient experiencing AL, but also their healthcare team, and medical system more broadly. The pressing need to mitigate these serious consequences supports further research in this area. With the recognition that ALs remain a reality of any surgical procedure that involves formation of an anastomosis, greater focus should be placed on exploring approaches for earlier detection—and by extension—treatment—of this severe and potentially life-threatening complication. While AL will no doubt remain a risk whenever surgical procedures involve formation of an anastomosis, new areas of research—such as those seeking to identify low-cost, non-invasive biomarkers that can be used as early leak predictors—are an important step towards mitigating the impacts of this devastating complication.
